# Venous-trapezium coupler anastomosis in a partially congested combined ALT/TFL-flap: A case report

**DOI:** 10.1016/j.jpra.2026.05.032

**Published:** 2026-05-22

**Authors:** S Gorji, P Stammer, A Dermietzel, T Hirsch, P Wiebringhaus

**Affiliations:** a Department of Plastic Surgery, University Hospital Muenster, Muenster, Germany; b Department of Plastic, Reconstructive and Aesthetic Surgery, Hand Surgery, Fachklinik Hornheide, Muenster, Germany; c Institute of Musculosceletal Medicine, University Hospital Muenster, Germany

**Keywords:** ALT-flap, TFL-flap, Venous congestion, Flap salvage technique, Intraosseous anastomosis, Complex limb reconstruction, Microsurgery

## Abstract

**Background:**

Reconstruction of large soft tissue defects with extensive vascular trauma remains a challenge. Intraosseous cannulation, commonly used in emergency medicine, may offer a novel solution for venous outflow. We report the use of the trapezium bone for venous drainage in a combined anterolateral thigh and tensor fasciae latae (ALT/TFL) flap reconstruction.

**Case:**

A patient presented with an extensive volar forearm defect, multifragmentary distal radius fracture, and transection of the radial artery, several flexor tendons and the superficial radial nerve from a circular saw injury. Initial management at an outside facility included radial artery ligation, nerve repair, external fixation, and negative pressure wound therapy (NPWT). A large ALT/TFL flap was harvested for definitive reconstruction. End-to-end anastomoses were performed to the proximal radial artery, two concomitant veins, and the superficial palmar branch distally. Due to lack of suitable distal veins, venous outflow was augmented by intraosseous cannulation of the trapezium using a 3–0 coupler, resolving distal flap congestion.

**Flap:**

perfusion was successfully restored with no need for further revision. Flap debulking was performed with power-assisted liposuction. The patient recovered uneventfully. At one-year follow-up, recovery sufficient for daily activities and work was achieved.

**Conclusion:**

Intraosseous venous drainage may offer a viable salvage technique when conventional venous outflow is not feasible. This novel approach may expand the reconstructive microsurgeon's armamentarium in complex trauma cases.

## Introduction

Reconstruction of traumatic defects presents a complex challenge often requiring advanced microsurgical techniques. The variability of accident circumstances leads to the uniqueness of each injury, necessitating individualized treatment approaches, as standardized protocols often fall short of addressing specific clinical needs.

An analysis of the German Trauma Registry reviewing 91.889 cases from 2021 to 2023, revealed that 28.8% of patients sustained injuries to the upper extremities.^1^ Major traumas with large defects frequently show the need for free flaps to achieve wound closure. A critical aspect of such procedures is the reliable reestablishment of arterial inflow and venous outflow to the defect site. Many studies have discussed alterations of the vascularity within the zone on injury, even to the extent of histological changes of vessels.^2^

One of the most frequent complications in reconstructive microsurgery is venous congestion of free flaps. A systematic review highlighted the limited evidence available regarding effective salvage techniques and emphasized the importance of timely and appropriate management.^3^ This scarcity of research is in contrast with the obvious relevancy of this topic, as venous thrombosis is one of the most common reasons for re-exploration or flap failure.^4^

As bone tissue is known to be highly vascularized, receiving 10 to 15% of the total cardiac output, it prompts the question of whether this system could support venous outflow in free flap reconstruction.^5^ Intraosseous (IO) access, a well-established technique in emergency medicine, similarly exploits the bone’s vascular network for rapid intravascular administration. This parallel invites exploration into the potential application of intraosseous pathways in reconstructive microsurgery, particularly in complex cases where conventional venous options are limited.

In this case report we present a novel surgical method of connecting the venous outflow of a combined anterolateral thigh and tensor fasciae latae (ALT/TFL) free flap to the venous system of the trapezium, using a 3–0 venous coupler. This could serve as a possible salvage option in situations where recipient veins are lacking or only insufficiently available.

## Case presentation

We report the case of a 54-year-old male machine operator who sustained a chainsaw injury to his right forearm, leading to a complex clinical scenario requiring comprehensive surgical intervention and management.

**Fig. 1:**
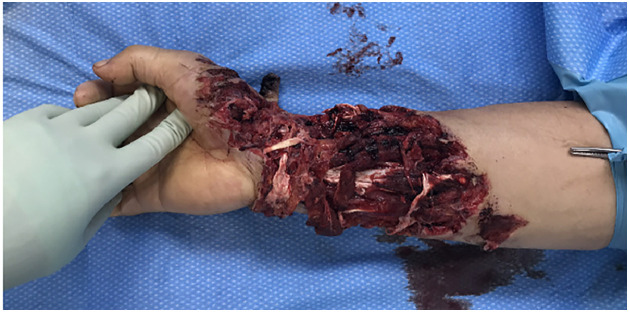
Initial presentation of trauma site.

The patient suffered a severe flexor injury to the distal right forearm, which included a large palmar soft tissue defect measuring approximately 27 × 15 cm (Fig. 1). The injury included extensive tendon injuries, namely the abductor pollicis longus and extensor pollicis brevis tendons. Additional transections involved the brachioradialis, flexor carpi radialis, flexor digitorum superficialis, and flexor carpi ulnaris tendons. Furthermore, a multifragmentary fracture of the distal radius with involvement of the first carpometacarpal joint was present. The radial artery was completely transected and subsequently ligated, while blood flow was preserved through the intact ulnar artery. Nerve injury included a transection of the superficial branch of the radial nerve.

**Fig. 2:**
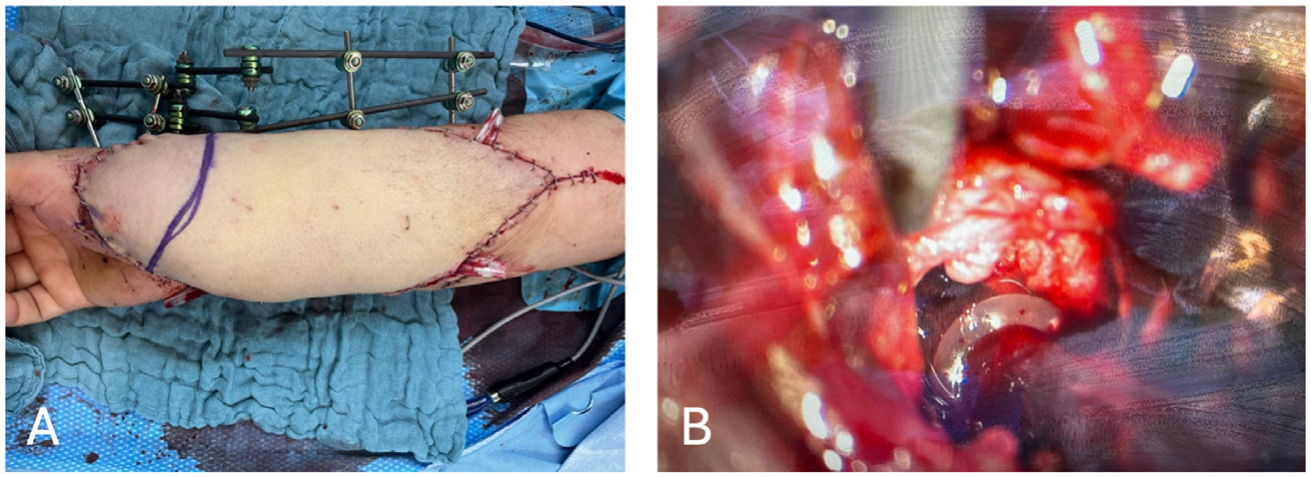
Intraoperatively the distal part of the ALT presented with signs of venous congestion (A), which was successfully treated, redirecting venous flow into the trapezium bone using a 3–0 venous coupler (B).

Initial surgical management included thorough debridement, necrosectomy, and application of negative pressure wound therapy (NPWT) to the distal forearm to optimize the wound bed. Reconstruction was planned using a combined ALT/TFL flap from the right thigh, measuring approximately 30 × 17 cm (Fig. 2A). During flap harvest, concerns arose regarding the adequacy of venous outflow for the combined flap due to the extent of the soft tissue injury and the limited availability of suitable recipient veins in the traumatized region. Therefore, a decision was made to delay the flap transfer. A NPWT was applied to the wound and the flap was left in situ and transferred tot he reciepient region on the forearm after one week during a second procedure. During definitive reconstruction, the ALT pedicle was anastomosed end-to-end to the proximal radial artery. Venous drainage of the ALT territory was established via two concomitant veins using 2.5-mm and 3.0-mm venous couplers. The TFL pedicle was subsequently anastomosed end-to-end to the superficial palmar branch of the radial artery distally. No additional suitable recipient vein was available in the operative field for the TFL venous outflow. An end-to-side anastomosis to the same recipient vein used for the ALT component was not feasible due to the spatial separation of the pedicles, while additional vein grafting was avoided because it would have required further anastomoses and prolonged operative time.

To address this challenge, an intraoperatively improvised technique was applied, where the venous outflow of the flap was directed into the spongiosa of the trapezium bone using a 3–0 venous coupler (Fig. 2B). This approach enabled effective venous drainage in the absence of peripheral recipient veins and contributed to the overall success of the flap salvage.

**Fig. 3:**
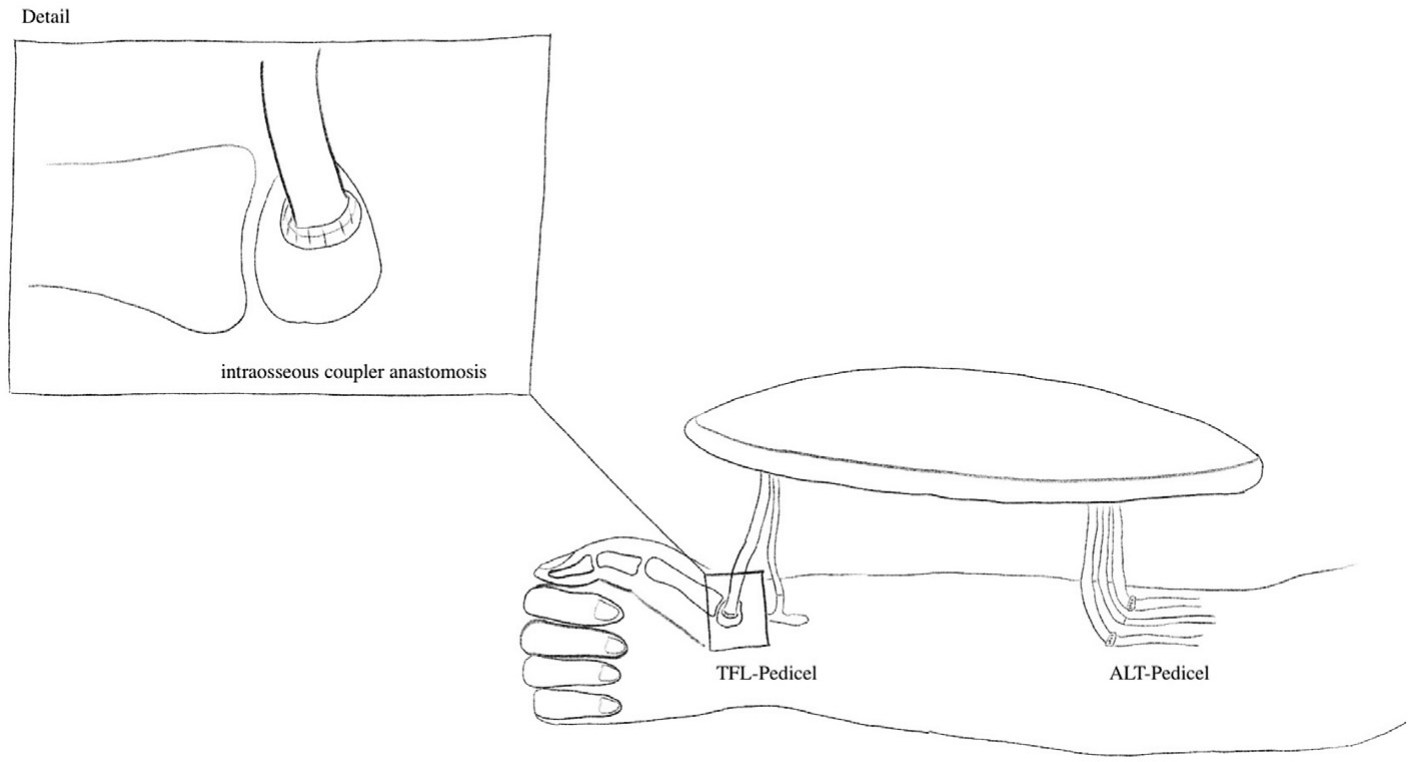
Illustration showing ALT/TFL flap anastomosis with detailed view on venous intraosseous coupler anastomosis in TFL-Pedicel.

**Fig. 4:**
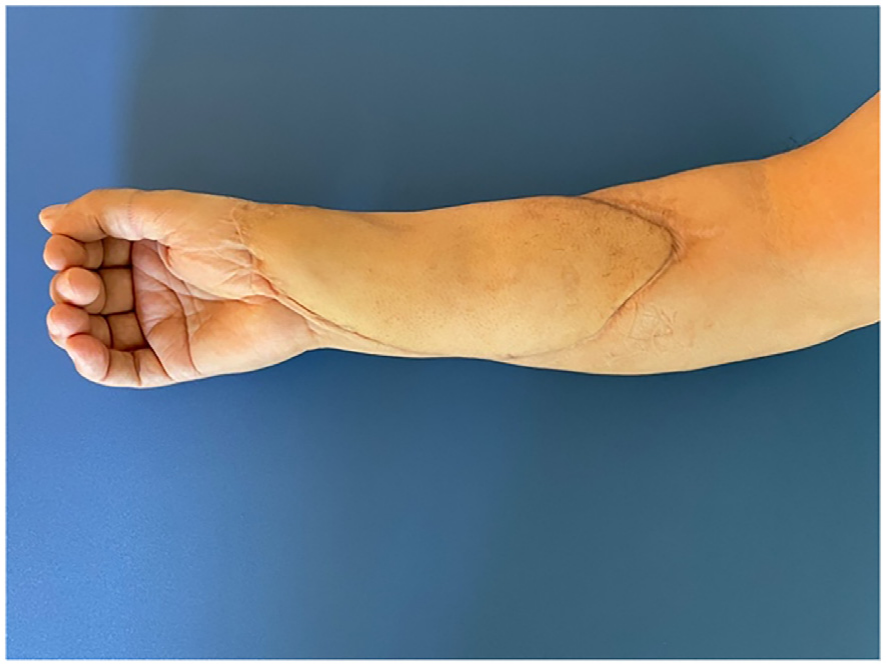
Postoperative result showing the combined ALT-TFL flap at one-year follow-up after flap debulking.

Postoperatively, the patient exhibited preserved motor and sensory function of the forearm, with consistent perfusion of the flap. The patient has successfully completed physiotherapy, achieving full recovery in function (Fig. 3).Fig. 4

## Discussion

The method described in this case has many similarities to the intraosseous access used commonly in emergency medicine. With the need for an intraosseus access being situations where venous access is unattainable, but urgently demanded, similar indications could apply to directing venous flow from flaps into bone sinusoids. A review article investigating 6525 adult patients found a combined complication rate following successful intraosseus catheter insertion of merely 4.6% indicating the safety of this principle.^6^

The described surgical technique presents multiple advantages. Directing the venous flow into bone tissue provides for flexible options, as this method is likely to extend beyond the trapezium as recipient bone structure for venous flow. Recent data supports the efficiency and safety of microvascular coupler systems in traumatic wrist revascularization, particularly in small-caliber vessels, although this evidence pertains specifically to coupler-assisted venous anastomosis rather than intraosseous outflow pathways and should therefore be interpreted with caution in this context.^7^

Currently there is no existing data on possible complications regarding the bone tissue, as for example osteonecrosis or osteomyelitis, while further radiographic evaluation would be required to verify this definitively. Although the patient of this case report did present with no further flap necrosis, it is unclear how the risk of venous thrombosis is affected by using bone sinusoids as recipient site. Bone fragments are likely to affect the local hemodynamic and create greater turbulences in blood flow, which could result in a higher risk of thrombosis. Furthermore, the coupler-device is not specifically manufactured for insertion in bone tissue. Additional modifications would be needed to optimally adjust the material to increase safety of this method.

Studies have pointed out the effectiveness of venous supercharging in treating venous congestion and the use of double venous anastomosis has been found to result in lower rates of thrombosis and flap-failure.^8^ The here described novel approach could be used in a similar way to give additional venous outflow in tissue areas where congestion is likely to occur.

In our case there was no suitable vein within the accessible range of the flap’s pedicle. An alternative method could have been an extension of this range using an autologous vein graft (e.g. saphenous vein). With no available palmar veins this graft would have needed sufficient length to reach dorsal veins of the forearm, increasing the risk of kinking and subsequent venous congestion. Additionally, the use of a vein graft would have further increased operating time, which was an important factor in the treatment of our patient. Here the time-saving benefit of connecting the venous flow to the intraosseous vascularity becomes evident. Another long known option in the treatment of venous congestion is leech therapy, which is however associated with risks of iatrogenic anemia and infection with the need of prophylactic antibiotics.^9^ Anatomical varieties in the vasculature of the bones, as Morsy et al. discussed distinct types of intraosseous vascular patterns could potentially have an impact on its eligibility as a recipient site for venous flow.^10^ In addition, it is unclear, whether other bones or bone types (long-bones / flat bones) would be more feasible for this approach.

## Conclusion

Intraosseous venous drainage, though commonly used in emergency medicine for fluid resuscitation, has not been previously described as a technique to support venous outflow in flap reconstruction. With this case report we present an additional salvage option to the treatment of venous congestion. Even cannulation of a small bone, in our case the trapezium, can provide an effective solution when traditional recipient veins were unavailable due to traumatic damage. The technique proved technically viable in resolving early signs of venous congestion. Its use may be particularly valuable in complex extremity injuries where recipient veins are thrombosed, damaged, or surgically inaccessible. Further investigation and reporting of similar cases will be necessary to validate this approach and define its indications, limitations, and long-term outcomes.

## Ethics approval

Ethical approval was not required for this single-patient case report in accordance with institutional and national guidelines.

## Availability of data and materials

All relevant clinical information is included within the manuscript. Additional data are available from the corresponding author upon reasonable request.

## Funding

No external funding was received for this work.

## Authors’ contributions

SG and PS collected the clinical data and drafted the manuscript. AD contributed to clinical assessment and data interpretation. TH and PW supervised the surgical aspects of the case and provided critical revisions to the manuscript. All authors read and approved the final manuscript.

## Declaration of generative AI and AI-assisted technologies in the manuscript preparation process

During the preparation of this work, the authors used ChatGPT (OpenAI) to assist with language editing and content organization. The authors reviewed and edited the content as necessary and take full responsibility for the final manuscript.

## Declaration of competing interest

The authors declare no competing interests.
